# Supplementary material for: Plasma microRNAs as a Potential Biomarker for Identification of Progressive Supranuclear Palsy
Source: Diagnostics (Basel). 2022 May 11;12(5):1204. doi: 10.3390/diagnostics12051204 (PMC9139891; doi:10.3390/diagnostics12051204)
Supplement: Supplementary file 1 [file diagnostics-12-01204-s001.zip › diagnostics-1714690-supplementary.pdf]

Article title: Plasma microRNAs as a potential biomarker for identification of progressive supranuclear palsy

Journal Name: Diagnostics, MDPI.

Palaniswamy Ramaswamy[1;#a], Rita Christopher[2] , Pramod Kumar Pal[1], Monojit Debnath[3], Ravi Yadav[1*]

[1]Department of Neurology, National Institute of Mental Health and Neuro Sciences (NIMHANS), Bengaluru, Karnataka, India

[2]Department of Neurochemistry, National Institute of Mental Health and Neuro Sciences (NIMHANS), Bengaluru, Karnataka, India

[3]Department of Human Genetics, National Institute of Mental Health and Neuro Sciences (NIMHANS), Bengaluru, Karnataka, India

[#a]Current address: Multi-Disciplinary Research Unit, Armed Forces Medical College, Pune, Maharashtra, India

***Corresponding author:**

E-mail: docravi20@yahoo.com

Table S1a Clinical characteristics of patients with PSP and healthy controls for initial miRNA screening

**Variable**	**Control (n=9)**	**Case (n=12)**	**P-value**
**Age, year (mean±SE)**	60.9±1.7	61.6±1.6	0.77
**Gender, male/female**	6/3	8/4	0.61
**Disease duration, year (mean±SE)**	-	2.4±0.37	
**PSPRS (mean±SD)**	-	40.8±1.06	
**Hypertension (%)**	0/9	0/12	
**Diabetes (%)**	0/9	0/12	
**Smoking (%)**	0/9	0/12	
**Alcohol (%)**	0/9	0/12	

Table S1b Demographic and clinical variables of patients with PSP and control subjects for validation

Variable	Controls (n=17)	Cases (n=18)	P-value
Age, year (mean±SE)	57.8±1.4	60.11±1.6	0.29
Gender, male/female	10/7	14/4	0.12
Disease duration, year (mean±SE)	-	2.37±1.17	
Hypertension (%)	4/17	3/18	
Diabetes (%)	2/17	3/18	
Smoking (%)	1/17	1/18	
Alcohol (%)	0/17	1/18	

N=number; S.E= standard error; SD=standard deviation; PSP= progressive supranuclear palsy; PSPRS= PSP rating scale.

Table S2 Plasma qPCR miRNAs profiling results with >1.5 or <1.5 fold change

miRNAs	Average Delta cq Case	Average Delta cq Control	Delta delta cq (case– control)	Fold change
hsa-miR-33a-5p	-1.49	-0.46	-1.03	2.04
hsa-miR-99a-5p	1.48	2.52	-1.04	2.06
hsa-miR-19b-3p	-4.50	-3.48	-1.02	2.03
hsa-miR-154-5p	2.46	3.18	-0.72	1.66
hsa-miR-92b-3p	4.01	5.22	-1.21	2.32
hsa-miR-32-5p	2.00	3.19	-1.19	2.28
hsa-miR-133a-3p	2.00	2.72	-0.72	1.65
hsa-miR-130a-3p	-3.50	-2.78	-0.72	1.65
hsa-miR-29b-3p	0.02	1.23	-1.21	2.31
hsa-miR-136-5p	1.50	2.20	-0.7	1.63
hsa-miR-142-5p	-2.51	-1.79	-0.72	1.65
hsa-miR-100-5p	4.51	5.15	-0.64	1.56
hsa-miR-326	0.00	0.72	-0.72	1.64
hsa-miR-193a-5p	4.48	5.13	-0.65	1.56
hsa-miR-127-3p	1.98	2.69	-0.71	1.64
hsa-miR-130b-3p	0.08	1.79	-1.71	3.29
hsa-miR-423-3p	-2.53	-1.81	-0.72	1.65
hsa-miR-210-3p	3.11	5.66	-2.55	5.85
hsa-miR-374a-5p	-0.50	0.21	-0.71	1.64
hsa-miR-877-5p	2.99	4.18	-1.19	2.28
hsa-miR-136-3p	2.51	3.73	-1.22	2.34
hsa-miR-29c-3p	-2.49	-1.78	-0.71	1.64
hsa-miR-543	3.72	2.51	1.21	2.31
hsa-miR-106a-5p	-2.50	-3.48	0.98	-1.96
hsa-miR-145-5p	-0.51	-1.47	0.96	-1.96
hsa-miR-424-5p	-0.49	-1.46	0.97	-1.96
hsa-miR-18b-5p	0.15	-1.32	1.47	-2.78
hsa-miR-223-5p	3.50	2.71	0.79	-1.73
has-miR-16-5p	-6.52	-6.48	-0.04	1.03

Table S3 Locked Nucleic Acid (LNA) microRNAs primers

miRNA	Mature miRNA sequence	Qiagen product number
hsa-miR-23a-3p	5'AUCACAUUGCCAGGGAUUUCC	YP00204772
hsa-miR-451a	5'AAACCGUUACCAUUACUGAGUU	YP02119305
hsa- miR-19b-3p	5'UGUGCAAAUCCAUGCAAAACUGA	YP00204450
hsa-miR-33a-5p	5'GUGCAUUGUAGUUGCAUUGCA	YP00205690
hsa-miR-130b-3p	5'CAGUGCAAUGAUGAAAGGGCAU	YP00204317
hsa-miR-136-3p	5'CAUCAUCGUCUCAAAUGAGUCU	YP00205503
hsa-miR-210-3p	5'CUGUGCGUGUGACAGCGGCUGA	YP00204333
hsa-miR-16-5p	5'UAGCAGCACGUAAAUAUUGGCG	YP00205702
RNA spike-in kit	UniSp2, UniSp6 & cel-miR-39-3p	339390

Table S4: Multiple KEGG pathways regulated by candidate microRNAs.

SNo.	KEGG pathway	p-value	#genes	#miRNAs
1	Fatty acid biosynthesis	1.89E-19	1	1
2	Endocytosis	7.82E-16	51	4
3	Hippo signaling pathway	3.60E-14	44	4
4	FoxO signaling pathway	6.76E-14	42	4
5	Protein processing in endoplasmic reticulum	9.87E-12	40	4
6	Estrogen signaling pathway	2.47E-11	24	4
7	Viral carcinogenesis	2.97E-11	29	3
8	p53 signaling pathway	4.23E-11	25	3
9	TGF-beta signaling pathway	9.09E-11	24	4
10	Proteoglycans in cancer	9.04E-09	35	4
11	Adrenergic signaling in cardiomyocytes	1.13E-08	26	4
12	Signaling pathways regulating pluripotency of stem cells	1.30E-08	26	3
13	AMPK signaling pathway	2.42E-08	34	3
14	Glioma	1.32E-07	17	3
15	Prostate cancer	1.92E-07	25	3
16	Chronic myeloid leukemia	2.30E-07	21	4
17	Colorectal cancer	5.94E-07	17	4
18	Hepatitis B	8.80E-07	29	4
19	Sphingolipid signaling pathway	9.84E-06	23	3
20	Endometrial cancer	1.44E-05	16	3
21	Pancreatic cancer	2.34E-05	17	4
22	Melanoma	2.47E-05	16	3
23	Pathways in cancer	7.34E-05	50	4

24	Cocaine addiction	7.81E-05	8	2
25	Non-small cell lung cancer	7.81E-05	14	3
26	PI3K-Akt signaling pathway	0.000225	46	3
27	Progesterone-mediated oocyte maturation	0.000246	20	3
28	Thyroid cancer	0.000302	7	3
29	Long-term depression	0.000406	12	3
30	Prolactin signaling pathway	0.000406	15	3
31	Acute myeloid leukemia	0.000867	14	3
32	Oocyte meiosis	0.001016	22	4
33	Chagas disease (American trypanosomiasis)	0.002782	20	4
34	Central carbon metabolism in cancer	0.005534	13	3
35	Bladder cancer	0.006369	10	3
36	Insulin signaling pathway	0.006369	23	4
37	Dopaminergic synapse	0.006829	23	4
38	Platelet activation	0.008042	19	4
39	Melanogenesis	0.008474	17	3
40	Fatty acid metabolism	0.008727	3	3
41	Hepatitis C	0.009513	22	3
42	Cholinergic synapse	0.011449	16	4
43	HTLV-I infection	0.012462	33	4
44	Oxytocin signaling pathway	0.028471	23	4
45	Toxoplasmosis	0.033598	17	4
46	Gap junction	0.037395	13	3
47	Cell cycle	0.038744	22	4
48	Renal cell carcinoma	0.044706	12	4

SI Table S5. Sorted KEGG pathways regulated by candidate microRNAs and associated target genes identified using three databases.

	FoxO signaling pathway	TGF-beta signaling pathway	Signaling pathways regulating pluripotency of stem cells	p53 signaling pathway	ErbB signaling pathway	Prolactin signaling pathway	Protein processing in endoplasmic reticulum	Cell cycle	Insulin signaling pathway	PI3K-Akt signaling pathway	Hippo signaling pathway	Wnt signaling pathway
hsa-miR-19b-3p	IRS2<br>CCNB1<br>STK4<br>PIK3CB<br>SETD7<br>MAPK14<br>CCND2<br>RAF1<br>CDKN1B<br>SMAD4<br>S1PR1<br>MAPK8<br>PRKAA1<br>CDKN1A<br>PTEN<br>TGFBR2<br>BCL2L11	INHBA<br>ACVR1<br>SMAD4<br>ZFYVE9<br>SMAD5<br>BAMBI<br>TGFBR2	GSK3B<br>FZD5<br>PIK3CB<br>MAPK14<br>RAF1<br>INHBA<br>ACVR1<br>SMAD4<br>SMAD5	ZMAT3<br>CCNB1<br>CCND2<br>CHEK1<br>CASP3<br>CDKN1A<br>PTEN	GSK3B<br>PIK3CB<br>RAF1<br>CDKN1B<br>MAPK8<br>CDKN1A	GSK3B<br>PIK3CB<br>MAPK14<br>CCND2<br>RAF1<br>MAPK8	DNAJA1<br>SAR1B<br>UBQLN2<br>SEC24C<br>MAPK8<br>HSP90B1<br>UBE2D3	GSK3B<br>CCNB1<br>CCNA2<br>CCND2<br>CDKN1B<br>CHEK1<br>SMAD4<br>CDKN1A	IRS2<br>GSK3B<br>PIK3CB<br>RAF1<br>CALM2<br>SREBF1<br>MAPK8<br>PRKAA1	GSK3B<br>PPP2R5E<br>PIK3CB<br>CCND2<br>RAF1<br>CDKN1B<br>HSP90B1<br>PRKAA1<br>CDKN1A<br>PTEN<br>BCL2L11	GSK3B<br>FZD5<br>CCND2<br>SMAD4<br>FRMD6<br>PARD6B<br>TGFBR2	GSK3B<br>FZD5<br>CCND2<br>SMAD4<br>MAPK8<br>BAMBI

hsa-miR-33a-5p	IRS2<br>CCNB1<br>SETD7<br>MAPK14<br>RAF1<br>CDKN1B<br>MAPK8<br>SOS1<br>BCL2L11	MYC<br>SMAD5<br>SP1	FZD5<br>MAPK14<br>RAF1<br>MYC<br>SMAD5	CCNB1<br>CASP3	RAF1<br>CDKN1B<br>MAPK8<br>MYC<br>SOS1	MAPK14<br>RAF1<br>MAPK8<br>SOS1	SAR1B<br>HERPUD1<br>UBQLN2<br>SEC24C<br>MAPK8<br>VCP<br>HSP90B1	CCNB1<br>CDKN1B<br>MYC	IRS2<br>RAF1<br>SREBF1<br>MAPK8<br>SOS1	PPP2R5E<br>RAF1<br>CDKN1B<br>MYC<br>SOS1<br>HSP90B1<br>BCL2L11	FZD5<br>MYC<br>PARD6B	GSK3B<br>CCND2<br>SMAD4<br>MYC<br>BAMBI
hsa-miR-130b-3p	CCNB1<br>STK4<br>PIK3CB<br>CCND2<br>SMAD4<br>S1PR1<br>SOS1<br>PRKAA1<br>CDKN1A<br>PTEN<br>TGFBR2<br>BCL2L11	INHBA<br>ACVR1<br>SMAD4<br>MYC<br>ZFYVE9<br>SMAD5<br>SP1<br>BAMBI<br>TGFBR2	GSK3B<br>PIK3CB<br>INHBA<br>ACVR1<br>SMAD4<br>MYC<br>SMAD5	ZMAT3<br>CCNB1<br>CCND2<br>CHEK1<br>CDKN1A<br>PTEN	GSK3B<br>PIK3CB<br>MYC<br>SOS1<br>CDKN1A	GSK3B<br>PIK3CB<br>CCND2<br>SOS1	DNAJA1<br>SAR1B<br>HERPUD1<br>CANX<br>VCP<br>UBE2D3	GSK3B<br>CCNB1<br>CCNA2<br>CCND2<br>CHEK1<br>SMAD4<br>MYC<br>CDKN1A	GSK3B<br>PIK3CB<br>CALM2<br>SOS1<br>PRKAA1	GSK3B<br>PIK3CB<br>CCND2<br>MYC<br>SOS1<br>PRKAA1<br>CDKN1A<br>PTEN<br>BCL2L11	GSK3B<br>CCND2<br>SMAD4<br>MYC<br>FRMD6<br>TGFBR2	FZD5<br>MAPK8<br>MYC
hsa-miR-136-3p	TGFB2	TGFB2					CANX		PPP1CB		TGFB2<br>PPP1CB	
